# A Whole-Brain Investigation of White Matter Microstructure in Adolescents with Conduct Disorder

**DOI:** 10.1371/journal.pone.0155475

**Published:** 2016-06-06

**Authors:** Sagari Sarkar, Flavio Dell’Acqua, Seán Froudist Walsh, Nigel Blackwood, Stephen Scott, Michael C. Craig, Quinton Deeley, Declan G. M. Murphy

**Affiliations:** 1 King’s College London, Sackler Institute for Translational Neurodevelopment and the Department of Forensic and Neurodevelopmental Sciences, Institute of Psychiatry, Psychology and Neuroscience, London, United Kingdom; 2 King’s College London, Department of Neuroimaging, Institute of Psychiatry, Psychology and Neuroscience, London, United Kingdom; 3 King’s College London, Natbrainlab, Department of Neuroimaging, Institute of Psychiatry, Psychology and Neuroscience, London, United Kingdom; 4 King’s College London, NIHR Biomedical Research Centre for Mental Health at South London and Maudsley NHS Foundation Trust and Institute of Psychiatry, Psychology and Neuroscience, London, United Kingdom; 5 Friedman Brain Institute, Icahn School of Medicine at Mount Sinai, New York, New York, United States of America; 6 King’s College London, Department of Child and Adolescent Psychiatry, Institute of Psychiatry, Psychology and Neuroscience, London, United Kingdom; University of North Carolina, UNITED STATES

## Abstract

**Background:**

The biological basis of severe antisocial behaviour in adolescents is poorly understood. We recently reported that adolescents with conduct disorder (CD) have significantly increased fractional anisotropy (FA) of the uncinate fasciculus (a white matter (WM) tract that connects the amygdala to the frontal lobe) compared to their non-CD peers. However, the extent of WM abnormality in other brain regions is currently unclear.

**Methods:**

We used tract-based spatial statistics to investigate whole brain WM microstructural organisation in 27 adolescent males with CD, and 21 non-CD controls. We also examined relationships between FA and behavioural measures. Groups did not differ significantly in age, ethnicity, or substance use history.

**Results:**

The CD group, compared to controls, had clusters of significantly greater FA in 7 brain regions corresponding to: 1) the bilateral inferior and superior cerebellar peduncles, corticopontocerebellar tract, posterior limb of internal capsule, and corticospinal tract; 2) right superior longitudinal fasciculus; and 3) left cerebellar WM. Severity of antisocial behavior and callous-unemotional symptoms were significantly correlated with FA in several of these regions across the total sample, but not in the CD or control groups alone.

**Conclusions:**

Adolescents with CD have significantly greater FA than controls in WM regions corresponding predominantly to the fronto-cerebellar circuit. There is preliminary evidence that variation in WM microstructure may be dimensionally related to behaviour problems in youngsters. These findings are consistent with the hypothesis that antisocial behaviour in some young people is associated with abnormalities in WM ‘connectivity’.

## Introduction

Conduct disorder (CD) is defined by a persistent display of antisocial behaviour such as deception, theft, vandalism and violence within a 6–12 month period in under-18s [1], and occurs in up to 16% of school aged children [2]. Children with severe CD cost society 10 times more to support into adulthood than those without CD [3]. Further, CD is strongly associated with other mental health problems (e.g. substance abuse [4] and mood disorders [5]), and antisocial personality disorder (ASPD) as adults [6]. It is likely that CD arises from a complex constellation of factors–and cannot be simply explained by any one putative social or biological causative agent alone. Nevertheless, despite the significant impact of CD on affected individuals and society as a whole, its biological determinants are still poorly understood.

Attempts to identify the neurobiological bases of CD using *in vivo* brain imaging have reported abnormalities in the structure [7–12] and function [13–18] of temporo-limbic and prefrontal brain regions. Hence there is increasing evidence that specific brain regions may be implicated in CD. However, brain regions do not act in isolation–they form part of large scale neural networks. Thus it is important to also examine the ‘connectivity’ of particular neural systems.

There is preliminary evidence that antisocial behaviour is associated with functional differences in the limbic-prefrontal network (that is associated with the generation of complex social and emotional behaviours) [15, 18–20]. The anatomical substrate for these functional differences in neural networks is unknown. However, we can now address this issue as the microstructural organisation of white matter (WM) tracts connecting neural systems can be indexed by measuring their fractional anisotropy (FA) using diffusion tensor magnetic resonance imaging (DT-MRI). FA is an index that quantifies directional differences in the diffusion of water molecules inside tissues. FA values range from 0 (perfectly isotropic diffusion) to 1 (perfectly anisotropic diffusion)—providing a proxy measure of tissue microstructural organization [21]. The microstructural basis for FA value is thought to lie with properties such as the organisation within and between fibres, axonal diameter, and myelination [22, 23].

We recently reported that adolescents with CD have increased FA of the uncinate fasciculus (UF) a major limbic-prefrontal WM connection, as compared to non-CD controls [24]. However, we used tractography based methods and so only examined a predefined tract of interest, and not whole brain. Therefore, the regional specificity of our previous finding (i.e. whether any additional tracts show abnormal microstructural diffusion properties in CD) is unknown. Tract-based spatial statistics (TBSS) is an automated method of whole-brain voxel based WM analysis [25].

Several studies of antisocial adults and children have examined whole brain WM using TBSS. For example, Sundram et al [26] reported reduced FA in the corpus callosum, corona radiata, inferior fronto-occipital fasciculus (IFOF), uncinate fasciculus, and internal capsule in males with ASPD. Similarly, Hoppenbrouwer et al’s [27] study of psychopathic offenders reported reduced FA in areas including the uncinate fasciculus, IFOF, and anterior thalamic radiation. However, studies of developmental samples have produced discrepant findings, reporting FA to be increased [28, 29], decreased [30] or no different [31] in adolescents with CD compared to controls.

The discrepancies between these studies may lie with methodological differences, including small sample size [31], and mixed sex samples [30, 31]. Specifically, in typical children and adolescents WM develops at a greater rate in males than females [32], so the increased FA we and others have observed in boys with CD may indicate an exaggeration of typical patterns of WM development [24, 28, 29, 33]. Furthermore, recent studies highlight important sex differences in the strength and distribution of WM ‘connectivity’ between sexes [34]. Our study sought to clarify the nature and extent of whole brain WM differences in CD using TBSS using a large, well characterised sample of adolescent males with CD and a group of non-CD controls.

## Methods and Materials

This study was approved by the Joint South London and Maudsley Research Ethics Committee (243/00).

### Participants

Twenty seven participants with CD aged between 12 and 19 years were recruited as part of a larger study (see [24]) from: (i) a Kings College London, Institute of Psychiatry database of adolescents with conduct problems; (ii) three Youth Offending Teams; (iii) five Pupil Referral Units (facilities providing education to children who cannot attend mainstream schools, e.g. following school exclusion); (iv) four youth projects; and (v) two mainstream educational institutions. A further twenty-one right handed males were recruited as controls from the general public, through schools and youth services (i.e. youth clubs, ‘Connexions’, and several youth charities) within the same geographical areas (deprived and inner city) as the CD group. Groups did not significantly differ in age, ethnicity, and self-reported history of alcohol or cannabis use. Furthermore, measures of current hyperactivity did not differ significantly between groups (see Table 1) and each group contained an equal number of boys with a prior diagnosis of ADHD (n = 2). To check for comorbid conditions, participants and parents were interviewed to ensure participants had no previous psychiatric diagnoses.

**Table 1:** Group characteristics. FSIQ—Full Scale Intelligence Quotient; SDQ–Strengths and Difficulties Questionnaire; APSD–Antisocial Process Screening Device; SD–standard deviation; #Excluding alcohol.

	Conduct disorder (n = 27) Mean (SD)	Healthy controls (n = 21) Mean (SD)	P value
Age in years	16 (2)	16 (2)	0.99
Mean FSIQ	99 (8)	110 (15)	0.01*
Conduct problems (SDQ)	6 (2)	3 (1)	0.00**
Hyperactivity (SDQ)	7 (2)	6 (2)	0.20
Emotional problems (SDQ)	2 (2)	3 (2)	0.15
Total problems (SDQ)	19 (5)	13 (4)	0.00**
Callous-unemotional traits (APSD)	7 (2)	5 (2)	0.00**
Total score (APSD)	25 (7)	18 (6)	0.00**
**Ethnicity (%)**			**Chi**^**2**^
White	52	62	0.49
Black/African-Caribbean	33	24	0.47
Other	15	14	0.96
**Substance use (%)**	**n = 20**	**n = 20**	**Chi**^**2**^
Cannabis—ever used	60	45	0.34
Cannabis–past month	30	35	0.74
Alcohol–ever used	75	95	0.08
Alcohol–past month	55	65	0.52
^*#*^Any other drug–ever used	44	43	0.09

*significant p value <0.05

**significant p value p<0.01

All study participants: satisfied MRI safety requirements and were medication free, did not have psychiatric or substance use disorders (other than CD, ADHD, or referrals for anger management), spoke English as their first language, and were right handed as assessed by the Edinburgh Handedness Inventory [35]. IQ was measured using the vocabulary and matrix reasoning subtests of the Wechsler Abbreviated Scale of Intelligence (WASI; [36]). We excluded individuals with IQ <80. Controls had a significantly higher IQ than CD individuals. Hence we co-varied for IQ in all subsequent analyses (see S1 Dataset).

### Measures

#### Questionnaires

Parent and self-report versions of the Strengths and Difficulties Questionnaire (SDQ; [37]) and Antisocial Process Screening Device (APSD; [38]) were administered. The SDQ was used to obtain conduct problem, emotional problem, and hyperactivity measures, while the APSD assessed callous-unemotional (CU) traits, narcissism, and impulsivity. Following methods of other groups, accepted subscales for both measures comprised the higher rater’s score for each item [14].

#### Interviews

CD and Oppositional defiant disorder (ODD) subsections of the Kiddie Schedule for Affective Disorders and Schizophrenia—Present and Lifetime version (K-SADS-PL; [39]) were used to obtain a research diagnosis of CD. Screening interviews for these disorders were administered to all participants, with those meeting criteria for CD or ODD given complete interviews for both disorders. No participants met criteria for ODD in the absence of CD. Finally, in order to assess psychopathic traits participants meeting CD criteria who additionally scored ≥ 20 on the APSD parent or self-report questionnaire were then interviewed using the Psychopathy Checklist Youth Version (PCL-YV; [40]). Scores of ≥ 20 were used to indicate the presence of psychopathic traits [41]. Interviews were conducted by a research psychologist (SSarkar) trained and supervised by a psychiatrist (QD). Additional information about antisocial behaviour was gathered from teachers, youth club workers, social workers and parents

Participants in the CD group had a history of serious aggressive and violent behaviour, including: robbery, burglary, grievous bodily harm, and sexual assault.

### Procedure

Full written informed consent was taken from participants, and additionally from a parent/guardian where boys were under 16 years old.

#### DT-MRI acquisition

Each DT-MRI image was acquired using a GE Signa HDx 3.0T MR scanner (General Electric, Waukshua, WI, USA), with actively shielded magnetic field gradients (maximum amplitude 40 mT m^-1^). The body coil was used for RF transmission, and an 8 channel head coil for signal reception, allowing a parallel imaging (ASSET) speed up factor of two. Head movement was minimised by fitting extra padding beside participants’ heads. Each volume was acquired using a multi-slice peripherally-gated doubly refocused spin echo EPI sequence, optimised for precise measurement of the diffusion tensor in parenchyma, from 60 contiguous near-axial slice locations with a voxel size of 1.875 x 1.875 x 2.4 mm. The echo time was 104.5 ms while the effective repetition time varied between subjects in the range 12 and 20 RR intervals. Based on the recommendations of Jones *et al* [42], the maximum diffusion weighting was 1300 s mm^-2^, and at each slice location, 4 images were acquired with no diffusion gradients applied, together with 32 diffusion-weighted images in which gradient directions were uniformly distributed in space. The sequence ran for approximately 15 minutes.

#### DT-MRI data pre-processing

All data were first converted to NIFTI format and then each raw diffusion dataset underwent a full quality control check where all B_0_s and Diffusion Weighted volumes were visually inspected for image corruption, motion artefacts, and signal drop-out effects using the light-box function available inside fslview (fmrib Software Library, www.fmrib.ox.ac.uk/fsl). Datasets showing more than 2 motion artefacts in different volumes on the same slice were removed from the study. Datasets showing significant head movements (>1 cm) were removed. No participant data acquired in this study required removal due to motion artefacts. Data were eddy current and motion corrected using Explore-DTI [43]. The diffusion tensor was estimated following removal of outlier data (RESTORE function [44]), and FA and MD maps were generated. Full details are given elsewhere [42].

#### DT-MRI analysis and statistics

First, the FA maps were transformed into standard stereotactic space using a study specific template generated in FSL (www.fmrib.ox.ac.uk/fsl), which was the FA map most representative of all FA images within the sample. All FA maps were averaged into a mean FA map for the whole sample and an average skeleton was created onto which each participant’s aligned FA data was projected. Finally, TBSS (part of FSL [45]) was applied to diffusion data for voxelwise analysis of whole brain WM [25]. Age and FSIQ, by group, were included as covariates in the design matrices used in the analysis, and results were corrected for multiple comparisons by FSL. The relevant contrasts identified regions where: FA in CD>Controls; FA in Controls>CD; FA in CD positively correlated with age; and FA in controls positively correlated with age. Regions showing significant FA differences (with a threshold of p<0.05; corrected for multiple comparisons) between groups were identified with reference to WM atlases [46]. For later correlation analysis (with behavioural and age data) it was necessary to extract FA values from regions of interest using masks created in FSL using the John Hopkins University (JHU) WM atlas [47]. FA values were correlated with SDQ and APSD behavioural scores, using Spearman’s rho non-parametric correlations; correlations were Bonferroni corrected (see S1 Dataset).

## Results

### Between group analysis

**Fig 1:**
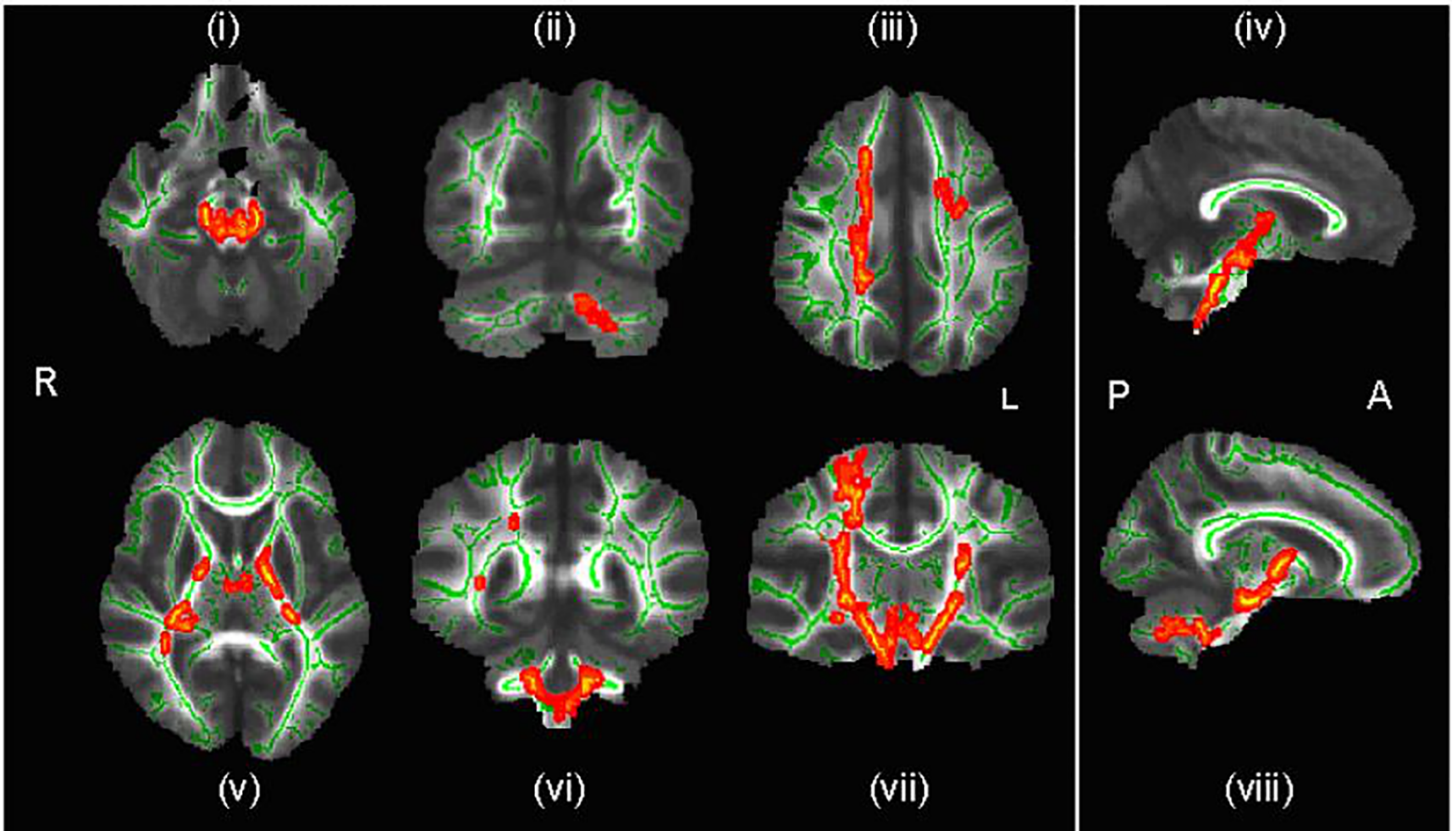
Regions of significantly increased fractional anisotropy in conduct disordered adolescents compared to healthy controls. Key: R-right; L- left; A-anterior; P-posterior; green indicates mean FA (fractional anisotropy) skeleton; red denotes areas of significantly greater (p < .05) FA in CD in: (i) bilateral superior cerebellar peduncle; (ii) left cerebellar white matter; (iii) right superior longitudinal fasciculus; (iv) bilateral corticopontocerebellar tract; (v) bilateral posterior limb of internal capsule; (vi) bilateral inferior cerebellar peduncle; (vii) bilateral corticospinal tract; (viii) bilateral corticopontocerebellar tract

The CD group had significantly greater FA as compared to non-CD controls in 7 regions: 1) bilaterally in the inferior and superior cerebellar peduncles, corticopontocerebellar tract, posterior limb of internal capsule, and corticospinal tract; 2) in the right superior longitudinal fasciculus; and 3) in left cerebellar WM (see Fig 1). Controls had no areas with significantly greater FA in comparison to the CD group.

### Behavioural relationships

There was no significant correlation between age and FA in either group, and there were no significant correlations between behaviour and FA within the CD or non-CD control groups alone.

**Fig 2:**
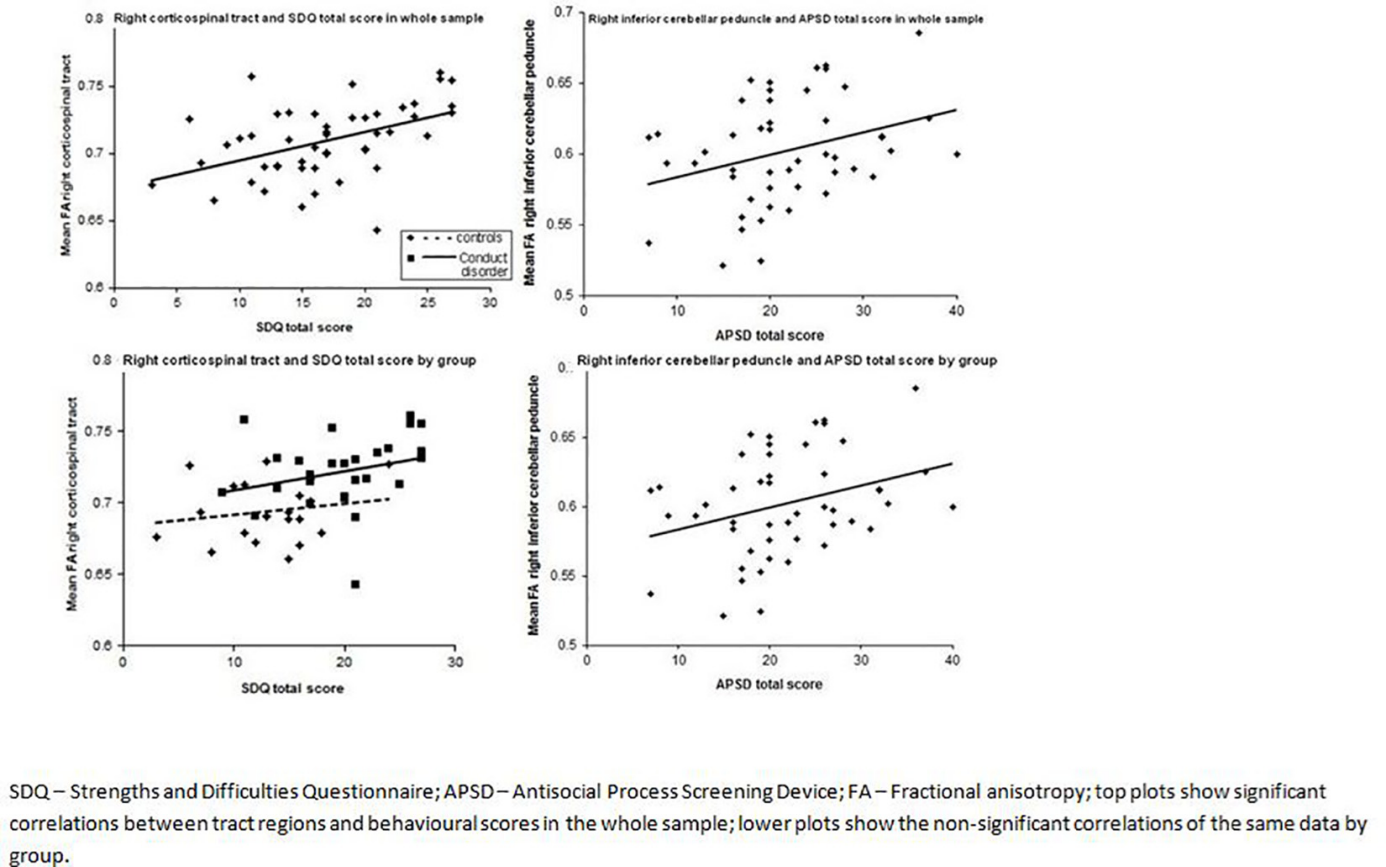
**Correlations between FA and behavioural measures in whole sample (above) and by group (below).** There were significant correlations between: 1) SDQ total problems and FA of the bilateral corticospinal tract, posterior limb of the internal capsule, superior cerebellar peduncle, and corticopontocerebellar tract; left cerebellar WM; and the right inferior cerebellar peduncle, and superior longitudinal fasciculus; 2) SDQ conduct problems and FA of the bilateral corticospinal tract, posterior limb of the internal capsule, superior cerebellar peduncle, inferior cerebellar peduncle, and corticopontocerebellar tract; and left cerebellar WM; 3) APSD total problems and FA of the bilateral inferior cerebellar peduncle; left cerebellar WM; and the right posterior limb of the internal capsule, superior longitudinal fasciculus, and corticopontocerebellar tract; 4) APSD callous-unemotional traits and FA of the right posterior limb of the internal capsule, superior longitudinal fasciculus, corticopontocerebellar tract; and left cerebellar WM; and 5) APSD impulsivity and the right inferior cerebellar peduncle.

In the whole sample, however, SDQ and APSD scores were positively correlated with FA values in all of the 7 regions that showed between group differences (see Table 2 and Fig 2).

**Table 2:** Correlations between SDQ and APSD scores and fractional anisotropy in whole sample. JHU–John Hopkins University; SDQ–Strengths and Difficulties Questionnaire; APSD–Antisocial Process Screening Device; CU–callous-unemotional; r–Spearman’s correlation coefficient; p–two-tailed significance level.

	SDQ				APSD					
JHU white matter atlas region	Total problems		Conduct problems		Total problems		CU traits		Impulsivity	
	r	p	r	p	r	p	r	p	r	p
**Corticospinal tract**										
Left	0.48	.00*	0.41	.00*	0.21	.15	0.19	.20	0.12	.40
Right	0.48	.00*	0.41	.00*	0.28	.06	0.23	.11	0.21	.14
**Internal capsule**										
Left posterior limb	0.33	.03	0.42	.00*	0.28	.06	0.34	.02	0.26	.08
Right posterior limb	0.43	.00*	0.41	.00*	0.30	.04	0.44	.00*	0.28	.05
**Cerebellar peduncle**										
Left superior	0.48	.00*	0.42	.00*	0.22	.14	0.20	.17	0.13	.40
Right superior	0.44	.00*	0.36	.01	0.26	.07	0.23	.12	0.18	.22
Left inferior	0.29	.05	0.36	.01	0.31	.03	0.24	.11	0.25	.09
Right inferior	0.38	.01	0.38	.01	0.31	.03	0.22	.14	0.37	.01
**Corticopontocerebellar tract**										
Left	0.43	.01	0.45	.00*	0.27	.06	0.29	.05	0.23	.12
Right	0.50	.00*	0.40	.01	0.30	.04	0.30	.04	0.27	.07
**Superior longitudinal fasciculus**										
Right	0.31	.03	0.25	.09	0.33	.02	0.32	.03	0.28	.06
**Cerebellar white matter**										
Left	0.35	.02	0.50	.00*	0.32	.03	0.36	.01	0.21	.15

*significant at p<0.05 after Bonferroni correction

## Discussion

We used whole-brain voxel-based DT-MRI to explore WM microstructural diffusion properties in a sample of adolescent boys with CD and a non-CD comparison group. There was significantly increased FA within regions corresponding with the trajectories of several WM tracts in CD boys compared to non-CD controls, namely: 1) bilaterally in the inferior and superior cerebellar peduncle, corticospinal tract, internal capsule (posterior limb), and corticopontocerebellar tract; 2) in the right superior longitudinal fasciculus; and 3) in the left cerebellar WM. There were no areas of significantly reduced FA in CD as compared to controls. There were significant positive correlations between FA and behavioural variables in all 7 tract regions showing between group differences in the total sample, but not in the CD or control groups alone.

Firstly, it is important to note that our analysis did not identify between group differences in limbic tracts such as the uncinate fasciculus (UF), as was the case in our group’s previous DT-MRI tractography study [24]. The reason for this may arise from the nature of TBSS analysis. The software used to perform the voxel-wise statistics (Randomise) sequentially compares every voxel to its corresponding voxel within each participant’s brain. Thus, differences seen using TBSS can be viewed as more locally defined (i.e. clusters of voxels) than tractography. In contrast, tractography can be used to detect more subtle differences along specific tracts. Thus, while we reported increased FA in the UF compared to the non-limbic control tracts in our prior study, this is likely not as pronounced in individual groups of voxels as the increases in FA found in the projection tracts observed in this study.

The increased FA we report contrasts with findings from studies of antisocial adults (e.g. [48]), possibly resulting from abnormal/precocious WM development in CD during childhood, which plateaus later in development [29]. This hypothesis is consistent with similar patterns of WM maturation observed in other specific neurodevelopmental disorders. For instance, structural MRI studies show that compared to neurotypical controls, people with autistic spectrum conditions (ASC) show increased WM volume in early childhood, but the opposite pattern (i.e. decreased volume) during adolescence [49]. This early acceleration also corresponds with DT-MRI research showing FA to be increased in specific brain regions (e.g. right inferior frontal gyrus and left occipital lobe [50]; corpus callosum and cingulum [51] in ASC. Future longitudinal studies should be undertaken to clarify whether this mechanism also occurs in CD.

However, that our study revealed FA to be increased, rather than decreased in the CD group is consistent with Zhang et al’s [28] study, and our group and others’ previous tractography study findings [24, 29]. Zhang et al reported increased FA in the corpus collosum and bilateral corona radiata. Although we did not find FA differences in the CC, the corticopontocerebellar and corticosprinal tracts in which we found FA increases, project through the corona radiata on their path from the cortex [52]. Therefore, while the precise location of FA increases differs between the two studies, both results may point to FA increases in the same tract. Finally, our results are in line with findings of increased FA in frontal WM associated with greater risk taking in young people [53]. The authors proposed that early WM maturation may stem from greater number/type of life experiences.

Although the precise significance of FA is not agreed on, it is regarded as a measure of inter- and intra-axonal properties, including the organisation within and between fibres, axonal diameter, and myelination [22, 23]. Therefore, the increased FA reported here most likely reflects differences in WM organisation or greater myelination of axonal tracts in CD. Alternatively, it is possible that rather than acceleration of the progressive process of myelination, increased FA in CD may reflect dysfunction in axonal pruning, the process by which surplus neuronal processes laid down in earlier developmental stages are removed, thereby refining information processing. However, it was not possible to directly test these two competing explanations in this study–but this issue could be addressed using *in vivo* myelin mapping [54].

The clusters of significantly increased FA we observed in the CD group correspond predominantly to projection tracts, which connect cortical and subcortical regions, including: the corticospinal and corticopontocerebellar tracts, and the posterior limb of the internal capsule (which carries the corticospinal tract fibres [55]). These WM tracts connect the cerebral cortex with the brainstem and the pons (which is further connected to the cerebellum). As well as its role in sleep and respiration, functions of the pons include sensory analysis, motor control, and the production of facial expressions. Animal studies demonstrate that pontine stimulation can provoke predatory attack [56]. This may explain how significant WM differences (which could cause differences in signal conduction through this region) may be associated with aggressive behaviour in CD.

The tracts within the prefrontal-thalamic-cerebellar circuit that we found to be abnormal in CD are also abnormal in both adolescents [57] and adults with alcohol use disorders [58–60]. These disorders, alongside early alcohol use, are predicted by childhood antisocial behaviour [61, 62]. Moreover, it has been suggested that these disorders arise from a common pathway [61]. Therefore, disruption to the prefrontal-thalamic-cerebellar WM pathways in CD may be related to, or underlie, the structural abnormalities seen in AUDs. Alternatively, the abnormalities we found in CD may be confounded by differences in alcohol use. However, this is unlikely to fully explain the differences we found, as the CD and control samples in our study did not significantly differ in self-reported alcohol and substance use. Thus, abnormalities in these tracts in a) young people with alcohol use disorders and b) young people with antisocial behaviour may reflect shared biological determinants affecting WM microstructure which (respectively) moderate risk of alcohol misuse and antisocial behaviour.

### Cerebellar peduncle

We also found between group differences in FA in the superior and inferior cerebellar peduncles. These fibre bundles are composed of efferent projections to the thalamus (superior cerebellar peduncle) or afferent projections from the spinal cord (inferior cerebellar peduncle). The major input to the cerebellar peduncle originates from the prefrontal, as opposed to the motor, cortex (PFC) [63], and this input is received in the most part via the corticopontine tract [64]. Thus, FA differences in these areas may indicate abnormal microstructure within the WM circuit connecting the PFC to the pons and cerebellum in boys with CD. The cause of this is unknown–but may stem from abnormal input from the PFC–as a number of prior neuroimaging studies have reported that CD individuals have significant differences in the anatomy and function of this brain region (e.g. [11, 41]). However, the current data cannot determine whether abnormality of the PFC is primary or secondary to differences in this tract. Longitudinal studies are required to elucidate this issue.

Evidence that increased FA of the cerebellar peduncle may be relevant to the generation of antisocial behaviour comes from patients with damage to PFC-cerebellar circuits. These individuals display some of the same deficits observed in antisocial children and adults. (i.e. emotional processing difficulties [65–67] and deficits of conditional associative learning [68]). Further, abnormal microstructural organization of cerebellar tracts has been reported in other neurodevelopmental disorders with differences in social function; e.g. in people with Autism Spectrum Disorders and schizophrenia [69, 70]. Thus, we do not suggest that abnormalities in PFC-cerebellar connections are specific to CD. Rather, they may underlie some aspects of social cognition deficits in a number of neurodevelopmental disorders. Nevertheless, these studies of other disorders only reported abnormality in selective tracts within the cortical-cerebellar-thalamic-cortical network, or of increased FA in some tracts but decreases in others. For example, reduced FA of the superior cerebellar peduncle was reported in Asperger syndrome, in the absence of deficits in cerebellar input pathways [69]. Further, in people with schizophrenia, increased FA of the superior cerebellar peduncle was found alongside reduced FA of the middle cerebellar peduncle, which contains afferent fibres [70]. In contrast, we found that individuals with CD have uniformly increased FA in all major cortical-cerebellar connections as compared with controls (i.e. the cerebellar peduncle, corticopontocerebellar tract, corticospinal tract, cerebellar WM). This suggests that a more generalized cortico-cerebellar ‘dysconnectivity’ (i.e. ‘abnormal functional integration of brain processes’ [71]) may underlie the emotional and behavioural deficits that characterise CD. Several studies have reported that young people with CD have concurrent abnormality in both prefrontal *and* cerebellar anatomy [8, 11, 12] or function [16, 18]. This suggests that both these regions may be abnormal in CD and that it is abnormal *interconnection* between these two regions that is of importance. Taken together, this study and that of others support the conjecture that boys with CD have abnormal cortico-cerebellar ‘connectivity’. It is unknown, however, which comes first (i.e. differences in brain anatomy or function, or behaviour).

### Corticospinal tract

Individuals with CD also had increased FA in the corticospinal tract. This tract contains predominantly motor axons, yet some prior work also suggests that it may be involved in emotion processing. For example, others reported [72], using transcranial magnetic stimulation, that in healthy controls threat signals, such as fearful facial expressions, increased the motor evoked potential of the corticospinal tract significantly more than positive or neutral faces [72]. This suggests that the corticospinal tract interacts closely with the limbic system to coordinate the motor response to threat-related stimuli. In this context, the increased FA of this tract detected by us in CD may indicate abnormality in responding to such cues. This suggestion fits with reports of abnormal neural responsivity to fearful faces and affective stimuli in both children with CD/CU traits [14–17], and adults with ASPD and psychopathy [73–75]. Future studies could investigate this relationship through correlating emotion processing measures, evoked potentials, and FA of the corticospinal tract.

### Superior longitudinal fasciculus

The area showing increased FA in CD corresponded with one of the subsections of the superior longitudinal fasciculus (SLF1), which connects the dorsal and medial parietal lobe with the dorsal and medial frontal lobe [76]. The precise function of this tract is not clear, as *in vivo* investigation of its anatomical characteristics has not long been possible [77]. However, the SLF1 is associated with the updating of verbal, as well as spatial, information; and deficits in verbal intelligence are noted in children with CD [78]. Thus, increased FA in this tract in CD may interfere with normal information processing and manifest as a verbal cognitive deficit. Investigations using DT-MRI tractography to correlate SFL1 integrity with verbal IQ scores may verify this.

### Behavioural relationships

In addition to the between group differences in FA, our behavioural analysis suggests that the regions in which greater FA was reported in CD may contribute towards the generation of the emotional and behavioural features of this disorder. While antisocial behaviour measures and FA were not correlated within the CD group, there was a significant positive correlation in the sample as a whole. It is possible that this correlation is caused by the fact that we merged data from two different sources–and so is ‘driven’ simply by between group differences and does not reflect a true dimensional (as opposed to categorical) relationship. In contrast, the regions of increased FA in CD may be dimensionally associated with antisocial behaviour across all adolescents, but the small sample size in this study may have prevented the correlations within each group from reaching significance. Preliminary support for this suggestion is provided by Fig 2, which exemplifies the distribution of SDQ and APSD scores against FA. This fits with the idea that conduct problems may fall along a continuum, and that the relationship between differences in brain and antisocial personality traits should be viewed as dimensional, rather than categorical [79].

Alternatively, however, it is possible that instead of FA differences underlying behavioural differences, it is the behavioural differences themselves that drive differences in WM microstructure. Support for this suggestion comes from studies investigating how social and emotional experience can modulate FA. For example, children who experienced severe deprivation in childhood had significantly reduced FA of the left uncinate fasciculus as compared to controls [80]. Similarly, others reported that young adults exposed to high levels of parental verbal abuse in childhood have significantly decreased FA in two left hemisphere limbic tracts (cingulum and fornix) and the arcuate fasciculus [81]. Conversely, increased FA appears can result from experience, such as after extensive practice of piano playing [82], meditation [83], and possibly from activity-dependent myelination resulting from social experiences [84]. Together with evidence of increased FA in high risk taking adolescents [53], it could be argued that the increased FA observed in CD may reflect increased myelination occurring as a result of these adolescents experiencing greater numbers/types of life experiences at an earlier age than their typical peers.

Therefore, the differences in tract integrity observed in developmental psychopathologies, such as in our study, most likely arise through a complex mixture of neurobiological, environmental and social factors. Future studies of WM maturation should consider (for example) the relative contribution of social and environmental variables in order to evaluate the potential relevance of early interventions to moderate or prevent the course of developmental disorders.

### Limitations

First, our groups showed a significant difference in mean FSIQ. Low IQ relative to the general population is characteristic of antisocial disorders [85, 86], so that cases and controls who are otherwise well matched may nevertheless significantly differ in IQ. We co-varied for FSIQ during our TBSS analyses to increase confidence that between group differences in FA are due to differences in conduct problems rather than IQ. Equally, there does not appear to be a simple relationship between IQ and FA in these tracts in adolescent boys (e.g. [87]).

Second, we did not find any correlations between FA and age in either group. This is in contrast to evidence of increased FA in adolescents who score highly on a scale of ‘risk taking’ [53]. A difference in sample size may explain these discrepant findings; therefore, future studies with larger sample sizes may reveal a relationship between age and WM in CD.

Third, it should be noted that methodological factors may have contributed to the increased FA values we detected. For example, DT-MRI derived outputs do not take into account complex WM organisation i.e. ‘crossing fibres’ [88, 89]. In the presence of multiple fibre orientations within the same voxel, the diffusion tensor provides only an average description of the real diffusion and this could make the interpretation of FA changes more difficult. For example, within the right corticospinal tract of Fig 1 (vii) there is an area that shows no significant FA difference flanked by two areas that do (corresponding to the corticospinal tract). It is possible either that the area showing no FA difference does not differ between groups, or, alternatively, that the two areas show increased FA only because there are fewer fibres crossing these portions of the cortical spinal tract.

However, crossing fibres cannot explain the behavioural associations we observed in our data, as within both groups of subjects the same regions were associated with behavioural scores. Nevertheless, possible methods for overcoming this limitation could be considered in the future by using tract specific indices that are not/less affected by the presence of crossing fibres. (see [90, 91]), and using the latest methods of dealing with this issue [92]

Fourth, while our groups did not significantly differ in self-reported alcohol and cannabis use it is widely noted that self-reported measures may be inaccurate. Furthermore, alcohol and substance use variables were coded for in a binary manner, which did not permit us to further control for these variables in our analyses. Future studies would benefit from assessing substance use quantitatively and obtaining objective measures of alcohol/substance use (e.g. using analysis of hair samples). However, given that FA changes in white matter associated with alcohol misuse tend to be diffuse reductions that affect multiple (mainly frontal) tracts suggests that substance misuse alone is unlikely to be driving our findings [93].

Last, it is important to note that while this study has identified correlations (i.e. associations) between brain structure and CD, it is not possible to clearly ascribe *causation*, define potential biological pathways, or rule out the influence of other factors on these neurodevelopmental outcomes. For example, data were not available that pertained to childhood trauma, negative family experiences, illnesses, and other events/neurochemical mechanisms that may influence brain development throughout infancy and childhood.

In summary, this study examined whole brain WM in boys with CD. Adolescent males with CD have significantly greater FA than controls in WM regions corresponding to the fronto-cerebellar circuit. There is preliminary evidence that variation in WM microstructure may be dimensionally related to behaviour problems in some youngsters. These findings are consistent with the hypothesis that antisocial behaviour in young people is associated with abnormalities in WM ‘connectivity’ within the fronto-cerebellar network.

## Supporting Information

S1 Dataset (SAV)
